# Case report: Conversion therapy for advanced intrahepatic cholangiocarcinoma using PD-1 inhibitor plus S-1 and nab-paclitaxel

**DOI:** 10.3389/fonc.2022.935817

**Published:** 2022-07-28

**Authors:** Xiaocheng Li, Zhiyang Jiang, Yongjuan Wu, Wei Gong, Xiaofeng Liao, Xiaogang Li

**Affiliations:** ^1^ Department of General Surgery, Xiangyang Central Hospital, Affiliated Hospital of Hubei University of Arts and Science, Xiangyang, China; ^2^ School of Medicine, Wuhan University of Science and Technology, Wuhan, China; ^3^ Department of Interventional Radiology, Xiangyang Central Hospital, Affiliated Hospital of Hubei University of Arts and Science, Xiangyang, China; ^4^ Department of Oncology, Xiangyang Central Hospital, Affiliated Hospital of Hubei University of Arts and Science, Xiangyang, China; ^5^ Institute of Oncology; XiangYang Central Hospital, Hubei University of Arts and Science, Xiangyang, China

**Keywords:** intrahepatic cholangiocarcinoma, case report, conversion therapy, complete remission, immunotherapy combined therapy

## Abstract

Intrahepatic cholangiocarcinoma (iCCA) is a highly malignant hepatobiliary tumor with a high rate of advanced disease at initial presentation. Conversion into resectable iCCA is important for improving the prognosis. Immunotherapy-based regimens are being increasingly used for treating advanced iCCA in recent years. However, the use of combined chemotherapy and immunotherapy for conversion has rarely been reported. The aim of this report was to present the outcomes of a 52-year-old female patient with IIIB iCCA. The patient was treated with a programmed cell death protein-1 inhibitor plus S-1 and nab-paclitaxel. The postoperative histopathological results indicated pathologic complete response after six cycles of systematic treatment. The patient is currently disease-free for one year.

## Introduction

Intrahepatic cholangiocarcinoma (iCCA) is the second most common liver malignancy after hepatocellular carcinoma (1); it is a highly malignant hepatobiliary tumor with an increasing incidence (2). Most of cases of iCCA are diagnosed in advanced stages at presentation, with a median survival of less than one year. Most patients are therefore no longer eligible for radical surgery, and chemotherapy forms an important part of treatment.

First-line chemotherapy for advanced iCCA includes gemcitabine, a platinum derivative, nab-paclitaxel, and fluoropyrimidines (3). However, only a few chemotherapy studies on chemotherapy were designed for iCCA alone. The BILCAP study compared capecitabine with observation following resection in patients with biliary tract cancer patients following resection. The median overall survival was prolonged from 36 months to 53 months in the capecitabine group (4). The ABC-06 study randomly compared folinic acid, fluorouracil, and oxaliplatin chemotherapy (FOLFOX) plus active symptom control with active symptom control alone as a second-line treatment for biliary tract cancer patients following cisplatin and gemcitabine failure (5). The results indicated that the FOLFOX regimen improved the overall survival rate by 14.5% at 12 months. In recent years, programmed cell death protein-1 (PD-1) inhibitors have shown effectiveness in conversion therapy for advanced liver cancer (6–9). An increasing number of studies are reporting promising outcomes with immunotherapy plus chemotherapy or targeted therapy for advanced liver cancer (10–17). Research indicates that iCCA has a rich tumor stroma; this suggests that immunotherapy may offer benefits in this tumor (18, 19). However, the outcomes with immunotherapy have been found to be unsatisfactory (19). Different studies indicate that chemotherapy, and especially 5-FU analogues, could upregulate programmed cell death ligand-1 (PD-L1) expression in tumor tissue and enhance the therapeutic effect of immunotherapy (20–26). However, the clinical benefit of immunotherapy plus chemotherapy for advanced iCCA remains unclear.

**Figure 1:**
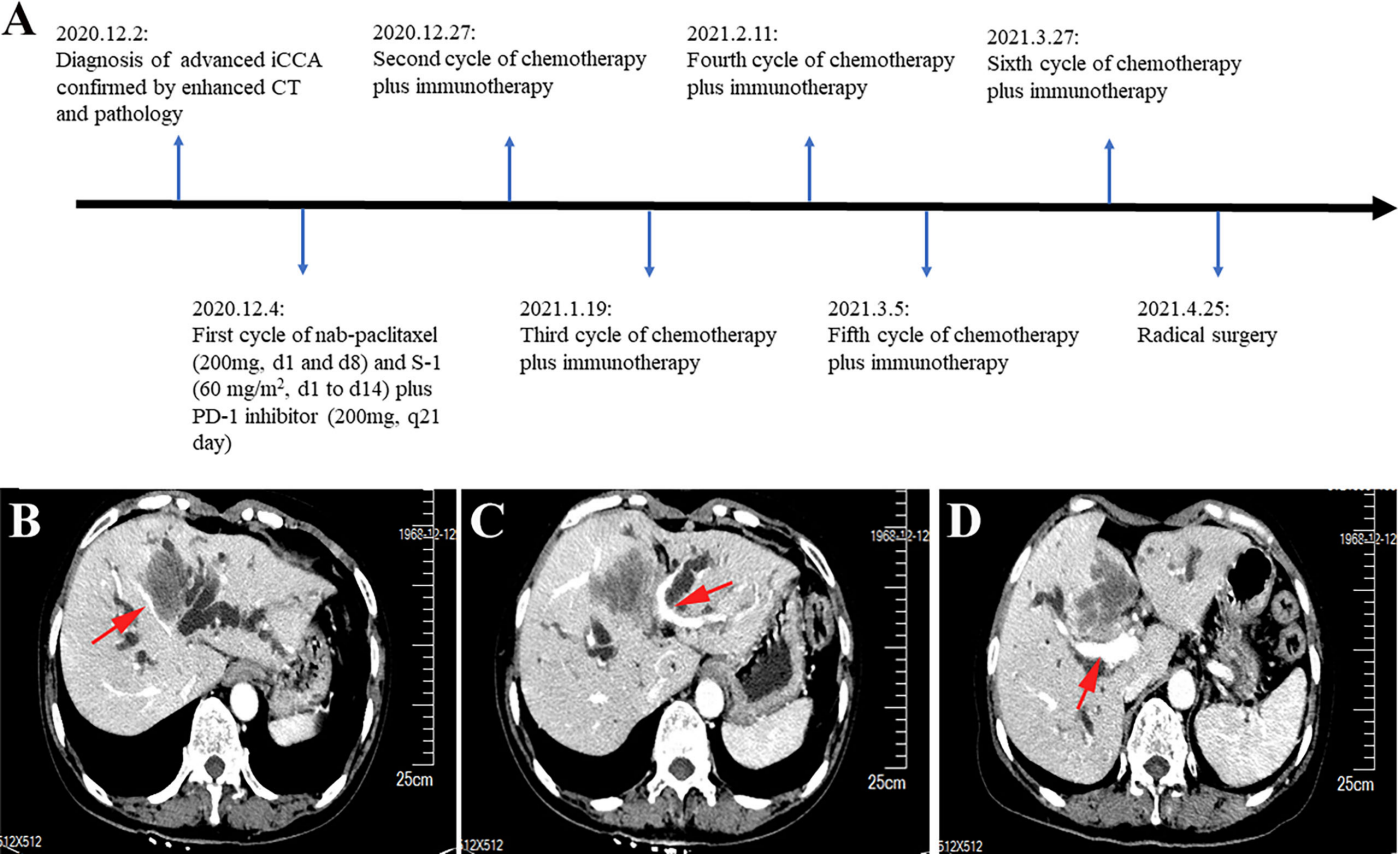
Timeline and enhanced computed tomography scan at the time of diagnosis. **(A)** Showing the course of initial diagnosis, medication, and surgery. **(B)** Showing a large mass in segment 4 of the liver invading the middle hepatic vein. **(C)** Showing the mass in segment 4 of the liver invading the umbilical portion of the left portal vein. **(D)** Showing the mass adjacent to the main trunk of the right portal vein.

This report presents the results of a new combined regimen with chemotherapy and immunotherapy for advanced iCCA, that offered successful conversion for radical resection. The postoperative specimen showed pathological complete response according to the Response Evaluation Criteria in Solid Tumors (version 1.1). The episode of care for this patient is summarized in **Figure 1A**.

## Case description

A 52-year-old woman was admitted to our hospital with jaundice for 6 days. She did not have a history of chronic hepatitis B or C infection. The Eastern Cooperative Oncology Group performance status score was 0. The total and direct bilirubin levels were 232.7 μmol/L and 186.1μmol/L, respectively, the alpha-fetoprotein level exceeded 1000 ng/ml, and the cancer antigen 19-9 level was 96.06 units/ml; the carcinoembryonic antigen level was within normal limits. An upper abdominal enhanced computed tomography (CT) scan showed a tumor measuring 5.8 cm in longest diameter in segment 4 of the liver. The portal vein phase indicated that the tumor had invaded the middle hepatic vein and the umbilical portion of the left portal vein and was adjacent to the main trunk of the right portal vein (**Figures 1B–D**). The tumor had also invaded the liver hilum, leading to biliary obstruction.

## Diagnostic assessment, therapeutic intervention, follow-up, and outcomes

**Figure 2:**
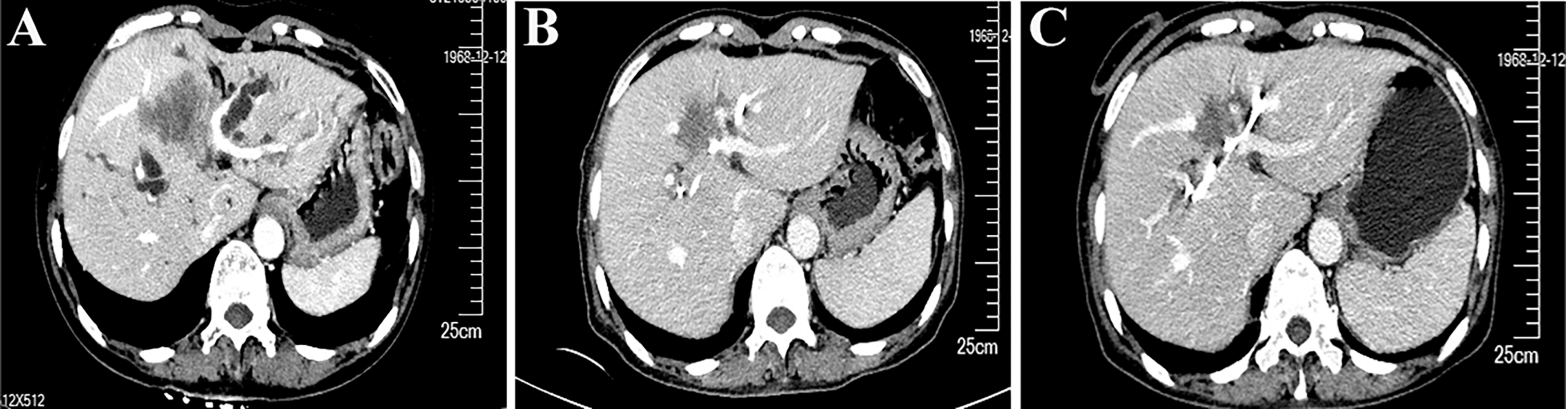
Cytologic examination and hematoxylin-eosin (HE) staining of liver tumor tissue from the needle biopsy and resected specimen. **(A)** Cytologic examination shows the presence of cancer cells (1000×). **(B)** HE staining (200×) of the liver tumor tissue from the needle biopsy. **(C)** HE staining (100×) shows only necrotic tissue in the resected tissue.

The patient underwent CT-guided percutaneous liver core biopsy and percutaneous transhepatic cholangiodrainage. Cytology confirmed the presence of cancer cells (**Figure 2A**); the results of immunohistochemical analysis were as follows: CK7 (+), CK19 (+), AFP (-), Hepatocyte (-), VILLIN (+), MOC-31 (+), GATA-3(-), CD34 (-), Glypican-3 (-), and Ki-67 labelling index: 70% (**Figure 2B**). According to the American Joint Committee on Cancer staging system, 8th edition, the patient was diagnosed with stage IIIB (T2N1M0) iCCA.

**Figure 3:**
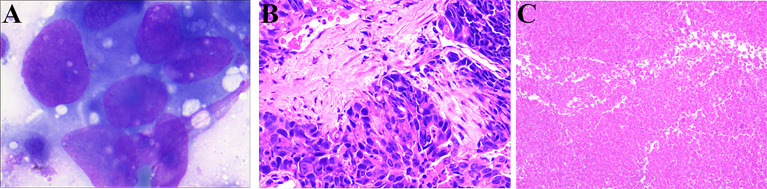
Enhanced computed tomography (CT) scans show that the cancer lesion changed over time. **(A)** CT results (2020.12.2) at the time of diagnosis. **(B)** CT results (2020.2.9) before the fourth cycle of treatment**. (C)** CT results (2020.4.23) before the radical surgery.

According to the opinion of a multidisciplinary team, she then received chemotherapy and immunotherapy after the total bilirubin had returned to the normal level following percutaneous transhepatic cholangiodrainage. After six cycles of treatment with nab-paclitaxel (200 mg, d1 and d8), S-1 (60 mg/m2, d1 to d14), and a PD-1 inhibitor (200 mg, q21 days), an enhanced CT scan showed that the longest diameter of the tumor shrank from 5.8 cm to 3.8 cm (**Figures 3A–C**). The treatment response was evaluated to be a partial response according to the revised Response Evaluation Criteria in Solid Tumors (version 1.1). The patient developed myelosuppression during the third cycle of chemotherapy and recovered on administration of growth factor injections. No immune-related adverse events were observed.

After surgical reassessment, the patient underwent hepatectomy (H2345’8’-B-MHV) (27), cholecystectomy, and biliodigestive anastomosis. Biliary leakage occurred on postoperative day seven and recovered after one month. No viable tumor cell was detected in the resected specimen; only necrotic tissue was detected, indicating a pathologic complete response after systematic treatment (**Figure 2C**). Additionally, the tissue in lymph node stations 7, 8, 9, and 12 were found to be entirely necrotic. The postoperative pathology results indicated down staging of the tumor to stage T1aN0M0 without perineural and vessel invasion; the resection margins indicated R0 resection status.

### Follow-up and outcome

The patient was discharged on postoperative day 31 and monitored every 3 months for recurrence at a local hospital by CT or magnetic resonance imaging. During the recent telephonic follow-up in May 2022, the patient informed that she was living a normal daily life without any symptoms. She had therefore achieved disease-free for one year and will undergo periodic radiographic follow-up.

## Discussion

To date, iCCA remains a challenging tumor without an effective treatment. Due to the highly aggressive nature of the cancer and its insidious onset, approximately 65% of cases are diagnosed in advanced stages with a median survival of less than 1 year. However, the median disease-free survival can rise up to three years after resection. Therefore, radical surgery after systemic treatment for unresectable iCCA has recently received increasing attention. The case in our study shows that conversion therapy for advanced iCCA can be achieved using a PD-1 inhibitor plus S-1 and nab-paclitaxel; this triplet regimen is safe and effective.

The patient initially presented with jaundice due to tumor compression. Preoperative biliary decompression has been traditionally performed in patients having malignant biliary obstruction with resectable tumors (28). However, growing evidence indicates that preoperative biliary decompression could increase postoperative complications (29–32). In patients with unresectable tumors, biliary decompression is necessary for improving liver function and facilitating subsequent chemotherapy. Complications associated with biliary decompression were not observed in our case.

The patient was evaluated *via* enhanced CT scans before treatment and was found to have N1 lymph node status. Lymph node dissection (LND) is recommended by the American Joint Committee on Cancer staging system, 8th edition, which suggests that at least six lymph nodes should be removed during LND (2). In this context, Kim et al. recommended that at least lymph node stations 8 and 12 should be dissected irrespective of the tumor location. Although LND is effective for evaluation of nodal status, studies are becoming increasingly skeptical about the benefits of LND for prognostication (33–35). To date, routine LND remains controversial; however, a multi-center study shows that selected patients with iCCA could benefit from LND (36).

Numerous studies on hepatocellular carcinoma have indicated that systemic conversion chemotherapy could make unresectable cases resectable (6, 7). However, conversion therapy for iCCA is relatively understudied. In a phase 2 clinical trial, nine of 41 (22%) patients with unresectable iCCA were successfully converted to surgically operable status using selective internal radiotherapy combined with chemotherapy (cisplatin and gemcitabine) (37). Riby et al. reported that 32 cases with initially unresectable iCCA in their cohort were resectable after administration of down staging chemotherapy with or without selective internal radiotherapy (38). In 2020, a French study tested FOLFIRINOX as first-line chemotherapy for advanced iCCA; 1 secondary resection was performed among 21 patients (39). In these studies, multiple chemotherapeutic agents were used to achieve good down staging. However, a combined chemotherapy regimen may be associated with severe adverse events. In another phase 2 trial, 60 patients with advanced biliary tract cancers were administered a regimen of nab-paclitaxel plus gemcitabine and cisplatin; nine (16%) patients withdrew owing to adverse events (40).

Although immunotherapy has demonstrated remarkable potency for different cancers, its efficacy in iCCA remains to be tested (41, 42). The KEYNOTE-158 study recruited 22 patients with cholangiocarcinoma who received immunotherapy; the overall response rate was 40.9% with a median progression free survival of 4.2 months (43). A phase II trial enrolled 54 patients with biliary tract cancer, including 32 cases of iCCA; the patients received at least one dose of immunotherapy and obtained a median progression free survival of 3.7 months (44).

Several ongoing studies are evaluating the efficacy of immunotherapy combined with gemcitabine with or without cisplatin; their results will be of particularly clinical value (18). In this case, we adopted a different combination of immunotherapy (PD-1 inhibitor) with S-1 plus nab-paclitaxel. S-1 is a prodrug of the active substance fluorouracil (5-FU); it can be preferentially converted to 5-FU in tumor cells (45). Studies indicate that 5-FU could induce PD-L1 expression in different cancers, including colorectal, gastric, and pancreatic cancer (20–26). Thus, we assumed that 5-FU may also upregulate PD-L1 expression in the tumor microenvironment of iCCA. The final pathological results support our hypothesis, as no active tumor cells were found in the specimen. A recent study reported a similar outcome to that of ours; in that study, a patient with advanced iCCA survived for over 16 months without progression after being treated with a PD-1 inhibitor plus capecitabine (46). Another group also successfully converted advanced iCCA to resectable status with PD-1 and tyrosine kinase inhibitors (47). These results indicate that immunotherapy may have a broader prospect in the conversion of advanced iCCA.

Although our results are promising, there are some limitations to this report. First, this report describes only one successful case; whether other patients are sensitive to this combined regimen is still unknown. A clinical trial with more patients will be needed to confirm our findings. Second, we could not test the expression level of PD-L1 due to complete necrosis of the tumor in the postoperative specimen. Further larger studies are needed to confirm whether 5-FU analogues may upregulate PD-L1 in iCCA.

### Conclusion

The findings from our case suggest that our regimen (S-1 and nab-paclitaxel plus PD-1 inhibitor) is suitable for converting advanced iCCA to resectable status; this provides a new treatment choice for this tumor. However, as this report describes only one case, studies on more patients are needed to verify its effectiveness in future.

## Patient perspective

When I got jaundice, I knew that something terrible happened to me. I was admitted to our local hospital and ordered a series of tests. After the results came out, the doctor asked me to transfer to the territorial central hospital. After I came to the territorial central hospital, the doctors kept encouraging me. While my jaundice improved, the doctor ordered chemotherapy and immunotherapy. At each post-treatment review, the doctor told me that the tumor was shrinking. It gave me great confidence in my treatment and made me forget the pain of chemotherapy. Finally, the doctor said to me that I was eligible for surgery. I felt a hope of rebirth. The operation was successful, and I am very grateful to the doctors. Until now, no tumor recurrence was found on postoperative monitoring. I am delighted with the treatment effect and feel confident for the future.

## Data availability statement

The original contributions presented in the study are included in the article/**Supplementary material**. Further inquiries can be directed to the corresponding author/s.

## Ethics statement

The studies involving human participants were reviewed and approved by the ethics committee of Xiangyang Central Hospital. The patients/participants provided their written informed consent to participate in this study.

## Author contributions

XCL, XGL, XFL, YJW, and WG conceived the idea for the article. XCL and ZYJ drafted the manuscript. XGL approved the final version of the manuscript. All authors contributed to the article and approved the submitted version.

## Funding

This work was supported by the Project of Hubei University of Arts and Science (Grant No.: XK2021042).

## Acknowledgments

The authors would like to thank all members of the study team and the patient and her family.

## Conflict of interest

The authors declare that the research was conducted in the absence of any commercial or financial relationships that could be construed as a potential conflict of interest.

## Publisher’s note

All claims expressed in this article are solely those of the authors and do not necessarily represent those of their affiliated organizations, or those of the publisher, the editors and the reviewers. Any product that may be evaluated in this article, or claim that may be made by its manufacturer, is not guaranteed or endorsed by the publisher.

## References

[1] Zhang H Yang T Wu M Shen F . Intrahepatic cholangiocarcinoma: Epidemiology, risk factors, diagnosis and surgical management. Cancer Lett (2016) 379(2):198–205. doi: 10.1016/j.canlet.2015.09.008 26409434

[2] Lee AJ Chun YS . Intrahepatic cholangiocarcinoma: The Ajcc/Uicc 8th edition updates. Chin Clin Oncol (2018) 7(5):52. doi: 10.21037/cco.2018.07.03 30180751

[3] Bupathi M Ahn DH Bekaii-Saab T . Therapeutic options for intrahepatic cholangiocarcinoma. Hepatobiliary Surg Nutr (2017) 6(2):91–100. doi: 10.21037/hbsn.2016.12.12 28503556 PMC5411274

[4] Primrose JN Fox RP Palmer DH Malik HZ Prasad R Mirza D . Capecitabine compared with observation in resected biliary tract cancer (Bilcap): A randomised, controlled, multicentre, phase 3 study. Lancet Oncol (2019) 20(5):663–73. doi: 10.1016/S1470-2045(18)30915-X 30922733

[5] Lamarca A Palmer DH Wasan HS Ross PJ Ma YT Arora A . Second-line folfox chemotherapy versus active symptom control for advanced biliary tract cancer (Abc-06): A phase 3, open-label, randomised, controlled trial. Lancet Oncol (2021) 22(5):690–701. doi: 10.1016/S1470-2045(21)00027-9 33798493 PMC8082275

[6] Petrowsky H Fritsch R Guckenberger M De Oliveira ML Dutkowski P Clavien PA . Modern therapeutic approaches for the treatment of malignant liver tumours. Nat Rev Gastroenterol Hepatol (2020) 17(12):755–72. doi: 10.1038/s41575-020-0314-8 32681074

[7] Song T Lang M Ren S Gan L Lu W . The past, present and future of conversion therapy for liver cancer. Am J Cancer Res (2021) 11(10):4711–24. PMC8569342 34765289

[8] Watanabe Y Osaki A Kimura K Yakubo S Takaku K Sato M . Unresectable primary hepatic adenosquamous carcinoma successfully treated with systemic and transcatheter hepatic arterial injection chemotherapies followed by conversion surgery: A case report and literature review. BMC Gastroenterol (2021) 21(1):491. doi: 10.1186/s12876-021-02070-3 34930149 PMC8686661

[9] Zhang T Merle P Wang H Zhao H Kudo M . Combination therapy for advanced hepatocellular carcinoma: Do we see the light at the end of the tunnel? Hepatobiliary Surg Nutr (2021) 10(2):180–92. doi: 10.21037/hbsn-2021-7 PMC8050575 33898559

[10] El-Khoueiry AB Sangro B Yau T Crocenzi TS Kudo M Hsu C . Nivolumab in patients with advanced hepatocellular carcinoma (Checkmate 040): An open-label, non-comparative, phase 1/2 dose escalation and expansion trial. Lancet (2017) 389(10088):2492–502. doi: 10.1016/S0140-6736(17)31046-2 PMC7539326 28434648

[11] Finn RS Ikeda M Zhu AX Sung MW Baron AD Kudo M . Phase ib study of lenvatinib plus pembrolizumab in patients with unresectable hepatocellular carcinoma. J Clin Oncol (2020) 38(26):2960–70. doi: 10.1200/JCO.20.00808 PMC7479760 32716739

[12] Hong JY Cho HJ Sa JK Liu X Ha SY Lee T . Hepatocellular carcinoma patients with high circulating cytotoxic T cells and intra-tumoral immune signature benefit from pembrolizumab: Results from a single-arm phase 2 trial. Genome Med (2022) 14(1):1. doi: 10.1186/s13073-021-00995-8 34986867 PMC8734300

[13] Li H Qin S Liu Y Chen Z Ren Z Xiong J . Camrelizumab combined with Folfox4 regimen as first-line therapy for advanced hepatocellular carcinomas: A Sub-cohort of a multicenter phase Ib/Ii study. Drug Des Devel Ther (2021) 15:1873–82. doi: 10.2147/DDDT.S304857 PMC8106453 33976538

[14] Marron TU Fiel MI Hamon P Fiaschi N Kim E Ward SC . Neoadjuvant cemiplimab for resectable hepatocellular carcinoma: A single-arm, open-label, phase 2 trial. Lancet Gastroenterol Hepatol (2022) 7(3):219–29. doi: 10.1016/S2468-1253(21)00385-X PMC9901534 35065058

[15] Ren Z Xu J Bai Y Xu A Cang S Du C . Sintilimab plus a bevacizumab biosimilar (Ibi305) versus sorafenib in unresectable hepatocellular carcinoma (Orient-32): A randomised, open-label, phase 2-3 study. Lancet Oncol (2021) 22(7):977–90. doi: 10.1016/S1470-2045(21)00252-7 34143971

[16] Xu J Shen J Gu S Zhang Y Wu L Wu J . Camrelizumab in combination with apatinib in patients with advanced hepatocellular carcinoma (Rescue): A nonrandomized, open-label, phase ii trial. Clin Cancer Res (2021) 27(4):1003–11. doi: 10.1158/1078-0432.CCR-20-2571 33087333

[17] Xu J Zhang Y Jia R Yue C Chang L Liu R . Anti-Pd-1 antibody shr-1210 combined with apatinib for advanced hepatocellular carcinoma, gastric, or esophagogastric junction cancer: An open-label, dose escalation and expansion study. Clin Cancer Res (2019) 25(2):515–23. doi: 10.1158/1078-0432.CCR-18-2484 30348638

[18] Kelley RK Bridgewater J Gores GJ Zhu AX . Systemic therapies for intrahepatic cholangiocarcinoma. J Hepatol (2020) 72(2):353–63. doi: 10.1016/j.jhep.2019.10.009 31954497

[19] Rizvi S Khan SA Hallemeier CL Kelley RK Gores GJ . Cholangiocarcinoma - evolving concepts and therapeutic strategies. Nat Rev Clin Oncol (2018) 15(2):95–111. doi: 10.1038/nrclinonc.2017.157 28994423 PMC5819599

[20] Del Re M Vivaldi C Rofi E Salani F Crucitta S Catanese S . Gemcitabine plus nab-paclitaxel induces pd-L1 mrna expression in plasma-derived microvesicles in pancreatic cancer. Cancers (2021) 13(15). doi: 10.3390/cancers13153738 PMC8345069 34359638

[21] Fukuoka E Yamashita K Tanaka T Sawada R Sugita Y Arimoto A . Neoadjuvant chemotherapy increases pd-L1 expression and Cd8(+) tumor-infiltrating lymphocytes in esophageal squamous cell carcinoma. Anticancer Res (2019) 39(8):4539–48. doi: 10.21873/anticanres.13631 31366557

[22] Lailler C Lamuraglia M Racine F Louandre C Godin C Chauffert B . DNA Damage response- and jak-dependent regulation of pd-L1 expression in head and neck squamous cell carcinoma (Hnscc) cells exposed to 5-fluorouracil (5-fu). Transl Oncol (2021) 14(8):101110. doi: 10.1016/j.tranon.2021.101110 33951601 PMC8111315

[23] Makowska A Meier S Shen L Busson P Baloche V Kontny U . Anti-Pd-1 antibody increases nk cell cytotoxicity towards nasopharyngeal carcinoma cells in the context of chemotherapy-induced upregulation of pd-1 and pd-L1. Cancer Immunol Immunother (2021) 70(2):323–36. doi: 10.1007/s00262-020-02681-x PMC7889576 32737537

[24] Petersen SH Kua LF Nakajima S Yong WP Kono K . Chemoradiation induces upregulation of immunogenic cell death-related molecules together with increased expression of pd-L1 and galectin-9 in gastric cancer. Sci Rep (2021) 11(1):12264. doi: 10.1038/s41598-021-91603-7 34112882 PMC8192931

[25] Van Der Kraak L Goel G Ramanan K Kaltenmeier C Zhang L Normolle DP . 5-fluorouracil upregulates cell surface B7-H1 (Pd-L1) expression in gastrointestinal cancers. J Immunother Cancer (2016) 4:65. doi: 10.1186/s40425-016-0163-8 27777774 PMC5067917

[26] Zhang M Fan Y Che X Hou K Zhang C Li C . 5-Fu-Induced upregulation of exosomal pd-L1 causes immunosuppression in advanced gastric cancer patients. Front Oncol (2020) 10:492. doi: 10.3389/fonc.2020.00492 32391259 PMC7188923

[27] Nagino M DeMatteo R Lang H Cherqui D Malago M Kawakatsu S . Proposal of a new comprehensive notation for hepatectomy: The "New world" terminology. Ann Surg (2021) 274(1):1–3. doi: 10.1097/SLA.0000000000004808 33630445

[28] Kagedan DJ Mosko JD Dixon ME Karanicolas PJ Wei AC Goyert N . Changes in preoperative endoscopic and percutaneous bile drainage in patients with periampullary cancer undergoing pancreaticoduodenectomy in Ontario: Effect on clinical practice of a randomized trial. Curr Oncol (2018) 25(5):e430–e5. doi: 10.3747/co.25.4007 PMC6209556 30464694

[29] Lai EC Lau SH Lau WY . The current status of preoperative biliary drainage for patients who receive pancreaticoduodenectomy for periampullary carcinoma: A comprehensive review. Surgeon (2014) 12(5):290–6. doi: 10.1016/j.surge.2014.02.004 24650759

[30] Chu J He S Ke Y Liu X Wang P Zhang W . The effect of preoperative biliary drainage with or without pancreatic stenting on complications after pancreatoduodenectomy: A retrospective cohort study. BioMed Res Int (2021) 2021:5572395. doi: 10.1155/2021/5572395 33997014 PMC8105100

[31] van der Gaag NA Rauws EA van Eijck CH Bruno MJ van der Harst E Kubben FJ . Preoperative biliary drainage for cancer of the head of the pancreas. N Engl J Med (2010) 362(2):129–37. doi: 10.1056/NEJMoa0903230 20071702

[32] Ramanathan R Borrebach J Tohme S Tsung A . Preoperative biliary drainage is associated with increased complications after liver resection for proximal cholangiocarcinoma. J Gastrointest Surg (2018) 22(11):1950–7. doi: 10.1007/s11605-018-3861-3 PMC6224307 29980975

[33] Hu H Xu G Du S Luo Z Zhao H Cai J . The role of lymph node dissection in intrahepatic cholangiocarcinoma: A multicenter retrospective study. BMC Surg (2021) 21(1):359. doi: 10.1186/s12893-021-01363-4 34627199 PMC8501613

[34] Morine Y Shimada M Utsunomiya T Imura S Ikemoto T Mori H . Clinical impact of lymph node dissection in surgery for peripheral-type intrahepatic cholangiocarcinoma. Surg Today (2012) 42(2):147–51. doi: 10.1007/s00595-011-0057-9 22124809

[35] Zhou R Lu D Li W Tan W Zhu S Chen X . Is lymph node dissection necessary for resectable intrahepatic cholangiocarcinoma? a systematic review and meta-analysis. HPB (Oxford) (2019) 21(7):784–92. doi: 10.1016/j.hpb.2018.12.011 30878490

[36] Ke Q Wang L Lin Z Lou J Zheng S Bi X . Prognostic value of lymph node dissection for intrahepatic cholangiocarcinoma patients with clinically negative lymph node metastasis: A multi-center study from China. Front Oncol (2021) 11:585808. doi: 10.3389/fonc.2021.585808 33777738 PMC7991319

[37] Edeline J Touchefeu Y Guiu B Farge O Tougeron D Baumgaertner I . Radioembolization plus chemotherapy for first-line treatment of locally advanced intrahepatic cholangiocarcinoma: A phase 2 clinical trial. JAMA Oncol (2020) 6(1):51–9. doi: 10.1001/jamaoncol.2019.3702 PMC6824230 31670746

[38] Riby D Mazzotta AD Bergeat D Verdure L Sulpice L Bourien H . Downstaging with radioembolization or chemotherapy for initially unresectable intrahepatic cholangiocarcinoma. Ann Surg Oncol (2020) 27(10):3729–37. doi: 10.1245/s10434-020-08486-7 32472411

[39] Ulusakarya A Karaboue A Ciacio O Pittau G Haydar M Biondani P . A retrospective study of patient-tailored folfirinox as a first-line chemotherapy for patients with advanced biliary tract cancer. BMC Cancer (2020) 20(1):515. doi: 10.1186/s12885-020-07004-y 32493242 PMC7268699

[40] Shroff RT Javle MM Xiao L Kaseb AO Varadhachary GR Wolff RA . Gemcitabine, cisplatin, and nab-paclitaxel for the treatment of advanced biliary tract cancers: A phase 2 clinical trial. JAMA Oncol (2019) 5(6):824–30. doi: 10.1001/jamaoncol.2019.0270 PMC6567834 30998813

[41] O'Donnell JS Teng MWL Smyth MJ . Cancer immunoediting and resistance to T cell-based immunotherapy. Nat Rev Clin Oncol (2019) 16(3):151–67. doi: 10.1038/s41571-018-0142-8 30523282

[42] Zappasodi R Merghoub T Wolchok JD . Emerging concepts for immune checkpoint blockade-based combination therapies. Cancer Cell (2018) 33(4):581–98. doi: 10.1016/j.ccell.2018.03.005 PMC5896787 29634946

[43] Marabelle A Le DT Ascierto PA Di Giacomo AM De Jesus-Acosta A Delord JP . Efficacy of pembrolizumab in patients with noncolorectal high microsatellite Instability/Mismatch repair-deficient cancer: Results from the phase ii keynote-158 study. J Clin Oncol (2020) 38(1):1–10. doi: 10.1200/JCO.19.02105 31682550 PMC8184060

[44] Kim RD Chung V Alese OB El-Rayes BF Li D Al-Toubah TE . A phase 2 multi-institutional study of nivolumab for patients with advanced refractory biliary tract cancer. JAMA Oncol (2020) 6(6):888–94. doi: 10.1001/jamaoncol.2020.0930 PMC7193528 32352498

[45] Miura K Shirasaka T Yamaue H Sasaki I . S-1 as a core anticancer fluoropyrimidine agent. Expert Opin Drug Deliv (2012) 9(3):273–86. doi: 10.1517/17425247.2012.652945 22235991

[46] Wang Z Zeng T Li Y Zhang D Yuan Z Huang M . Pd-1 inhibitors plus capecitabine as maintenance therapy for advanced intrahepatic cholangiocarcinoma: A case report and review of literature. Front Immunol (2021) 12:799822. doi: 10.3389/fimmu.2021.799822 35003124 PMC8739978

[47] Zhang Z Zhang W Wang H Hu B Wang Z Lu S . Successful treatment of advanced intrahepatic cholangiocarcinoma with a high tumor mutational burden and pd-L1 expression by pd-1 blockade combined with tyrosine kinase inhibitors: A case report. Front Immunol (2021) 12:744571. doi: 10.3389/fimmu.2021.744571 34603331 PMC8484748

